# Development of a High-Throughput Three-Dimensional Invasion Assay for Anti-Cancer Drug Discovery

**DOI:** 10.1371/journal.pone.0082811

**Published:** 2013-12-11

**Authors:** Nikki A. Evensen, Jian Li, Jie Yang, Xiaojun Yu, Nicole S. Sampson, Stanley Zucker, Jian Cao

**Affiliations:** 1 Department of Medicine/Cancer Prevention, Stony Brook University, Stony Brook, New York, United States of America; 2 Department of Preventive Medicine, Stony Brook University, Stony Brook, New York, United States of America; 3 Department of Tissue Engineering, Stevens Institute of Technology, Hoboken, New Jersey, United States of America; 4 Department of Chemistry, Stony Brook University, Stony Brook, New York, United States of America, and; 5 Department of Research, Veterans Affair Medical Center, Northport, New York, United States of America; University of South Alabama, United States of America

## Abstract

The lack of three-dimensional (3-D) high-throughput (HT) screening assays designed to identify anti-cancer invasion drugs is a major hurdle in reducing cancer-related mortality, with the key challenge being assay standardization. Presented is the development of a novel 3-D invasion assay with HT potential that involves surrounding cell-collagen spheres within collagen to create a 3-D environment through which cells can invade. Standardization was achieved by designing a tooled 96-well plate to create a precisely designated location for the cell-collagen spheres and by using dialdehyde dextran to inhibit collagen contraction, maintaining uniform size and shape. This permits automated readout for determination of the effect of inhibitory compounds on cancer cell invasion. Sensitivity was demonstrated by the ability to distinguish varying levels of invasiveness of cancer cell lines, and robustness was determined by calculating the Z-factor. A Z-factor of 0.65 was obtained by comparing the effects of DMSO and anti-β1-integrin antibody, an inhibitory reagent, on the invasion of Du145 cancer cells, suggesting this novel assay is suitable for large scale drug discovery. As proof of principle, the NCI Diversity Compound Library was screened against human invasive cancer cells. Nine compounds exhibiting high potency and low toxicity were identified, including DX-52-1, a compound previously reported to inhibit cell migration, a critical determinant of cancer invasion. The results indicate that this innovative HT platform is a simple, precise, and easy to replicate 3-D invasion assay for anti-cancer drug discovery.

## Introduction

Metastasis is the main cause of death in cancer patients and one of the most complex biological processes in human diseases[1]. A major challenge in the development of anti-cancer drugs aimed at preventing metastasis is the lack of effective tools for drug discovery. Drug discovery programs have primarily focused on target-based screening, which has resulted in new categories of pharmacologic agents, primarily tyrosine kinase inhibitors and monoclonal antibodies[2]. However, since most types of cancers develop numerous mutations during tumor progression[3], individual cancer cell clones often circumvent inhibitory compounds focused on single targeted molecules[4]. The failure to commercialize many publicly funded target-based drug discoveries is driving the need for novel approaches to expedite drug development for metastatic cancers. Thus, developing novel tools that can be used for identifying effective drugs targeting the invasive phenotype of cancer cells, a requirement at the early stage of metastasis[5], and the later stage of cancer recurrence due to self-seeding from micro/macro-metastasized tumors[6], is imperative in converting this life threatening disease to a chronic one. 

Effective targeting of cancer cell invasion requires an amenable technology to mimic *in vivo* conditions. Mounting evidence has demonstrated that three-dimensional (3-D) matrices for cancer cell culture more closely mimic *in vivo* conditions as compared to 2-D cell culture conditions. Therefore screening in 3-D culture conditions offers a higher predictive value for future clinical efficacy of potential drugs and allows for better optimization[7,8]. Current 3-D high-throughput (HT) screening assays are focused primarily on identifying compounds that inhibit tumor growth and proliferation [9–15]. Attempts have been made to develop 3-D invasion assays with HT capability[16–18]. However, the use of these assays for anti-cancer cell invasion drug discovery has been stymied due to the high cost of large-scale screening[16,17], a requirement for long incubation times (e.g., 3 to 5-day incubation periods) for endpoint readouts determined by low signal:background ratios[17], cumbersome procedures[16,18,19], and/or a lack of standardized and automated techniques for quantifying cell invasion[20]. 

Herein, we provide a novel collagen gel spheroid-based 3-D invasion HT screening tool for identifying drugs that show anti-invasion capabilities. By utilizing an innovatively tooled plate, along with dialdehyde dextran mixed with collagen to prevent contraction, this novel 3-D invasion assay permits standardization and automated readout, which are key requirements for HT screening. We provide evidence that our 3-D invasion assay is reproducible, effective, easy and rapid to perform, and sensitive enough to identify compounds that inhibit cancer cell invasion. The potential for these active hits to have a positive impact on patients with cancer by preventing dissemination supports the use of this assay in current drug discovery programs.

## Materials and Methods

### Materials

Type I collagen (acetic acid-extracted native type I collagen from rat tail tendon) and propidium iodide (PI) were purchased from BD Bioscience Discovery Labware. Recombinant tissue inhibitor of metalloproteinase-2 (TIMP-2) and anti-MT1-MMP hemopexin domain antibody were purchased from Chemicon International, Inc. RNAi-Ready pSIREN Retro-Q vector for specific gene silencing and pQCXIP retroviral vector for generation of stable cells were purchased from Clontech. Hoechst nuclear stain was purchased from Invitrogen. The anti-β1 integrin antibody was purchased from GE Healthcare. Staurosporine, paclitaxel, and dextran (Mw=500,000) were purchased from Sigma.

### Cell Lines and Treatment of Cells

Human fibrosarcoma HT-1080, human prostate cancer LNCaP, Du145, and PC3, human breast epithelial cancer MDA-MB-231 cell lines were purchased from ATCC. The LNCaP cells expressing Green Fluorescent Protein (GFP) or MT1-MMP-GFP chimeric cDNAs were previously described[21]. The cells were maintained in DMEM-high glucose or RPMI 1640 medium (Invitrogen) containing 10% FBS and 1% Pen/Strep. The SK-3^rd^ cells and mammosphere formation were previously described[22]. Briefly, these cells were cultured in ultra-low attachment dishes (Corning) in suspension with DMEM-F12 (Cellgro) medium supplemented with B27 (1:50, Invitrogen), EGF (20 ng/mL BD Biosciences), 0.4% bovine serum albumin (Sigma), and 4 μg/ml insulin (Sigma)[22]. 

### 3-D Cell Scattering Assay

This assay was performed as previously described[21]. Briefly, single cell suspensions of LNCaP cells (4x10^4^ cells/ml) expressing GFP or MT1-MMP-GFP were mixed with type I collagen (2.5 mg/ml final concentration). The cell-collagen gel cultures were allowed to solidify in 24-well plates. Following solidification, complete medium was added with or without indicated inhibitors. Cell scattering ability was monitored under a Nikon microscope over 6 days.

### 3-D Multi-cell Spheroid Invasion Assay

To generate cell aggregates, SK-3^rd^ cells (1000 cells/ml) were cultured in ultra-low adhesion culture dishes for 12 days. Spheres were then transferred to 24-well dishes containing type I collagen. The embedded spheroids were then cultured for 3 days under normoxic or hypoxic conditions. For hypoxic conditions, cells were incubated using a BioSpherix ProOx C21 set to 1% O_2_ and 5% CO_2_. For the multi-cell spheroid method utilizing glass microcarrier beads, the LNCaP MT1-MMP-GFP expressing cells were cultured with beads for 24 hours with shaking. The cell-bead aggregates were then transferred and embedded in type I collagen and cultured for an additional 3 days.

### 3-D Invasion Assay

Cancer cells (8x10^7^ cells/ml) were mixed with an equal volume of 3 mg/ml neutralized type I collagen on ice, followed by the addition of dialdehyde dextran solution (final 0.25% of total volume). The cell-collagen mixture was then dotted into the pits in the center of each well of a 96-well plate. The optimal size of the pit in each well is 2 mm in diameter and requires 4.18 mm^3^ (μl) [4/3πr³, r=1 mm] of cell-collagen mixture to create a spherical dot within the pit. After solidification of cell-collagen dots at 37°C, a layer of neutralized type I collagen (80 μl, 1.5 mg/ml final concentration) was added to cover the cell-collagen hemispheres. After a 10 minute incubation at 37 °C, the assembled cell invasion complex was covered with 80 μl of medium with added inhibitors or vehicle.

### Observation of Invaded Cells

Invaded cancer cells were stained with both Hoechst 33342 (25 μg/ml) for nuclear staining of total cells and PI (2.5 μg/ml) for staining of dead cells followed by extensive washing with PBS. Invasion of cancer cells was then examined using fluorescent microscopy. To automate the quantification of invaded cells, phase contrast and Hoechst images for the entire plate were obtained. A threshold from the phase contrast image of a control (untreated or vehicle treated) well of the 96-well plate was adjusted based on the difference in contrast between outside and inside of the pit to create two binary layers. The binary layer outside the pit was copied to form a region of interest (ROI), which was then applied to the Hoechst images. The invaded cells within the ROI were then automatically counted using object count. This was done using NIS-Elements Br 3.2 Software.

### Oxidation of Dextran to Form Dialdehyde Dextran

2.5 g of dextran was dissolved into 100 ml diH_2_O. Sodium periodate (NaIO_4_) (1.65 g) was added for 18 hours incubation, with agitation, at room temperature. To quench the reaction, 1 ml ethylene glycol was added. The solution was dialyzed with diH_2_O for 3 days, lyophilized, and reconstituted with diH_2_O to a final concentration of 2.5% dialdehyde dextran. 

### Protease Assay

Total cellular proteolytic activity was determined using a Fluorescent Detection Kit (Sigma). Briefly, cell lysates were incubated for 20 hours at 37°C with fluorescein isothiocyanate (FITC)-labeled casein substrate. Lysates were trichloroacetic acid (TCA) precipitated. Supernatants containing fragmented FITC-casein were then combined with assay buffer and their fluorescence intensity was measured with excitation at 485 nm and emission at 535 nm using a SpectraMax Gemini EM (Molecular Devices) fluorescent plate reader.

### Statistical Analyses

Student unpaired two sided t-test was used to assess differences with p values < 0.05 considered statistically significant. Pooled data were presented as mean ±SD for all analyses of three independent experiments. GraphPad Prism 5 software was used for this analysis.

## Results

### Limitations of Current Invasion Assays Hindering HT Screening Capabilities

**Figure 1:**
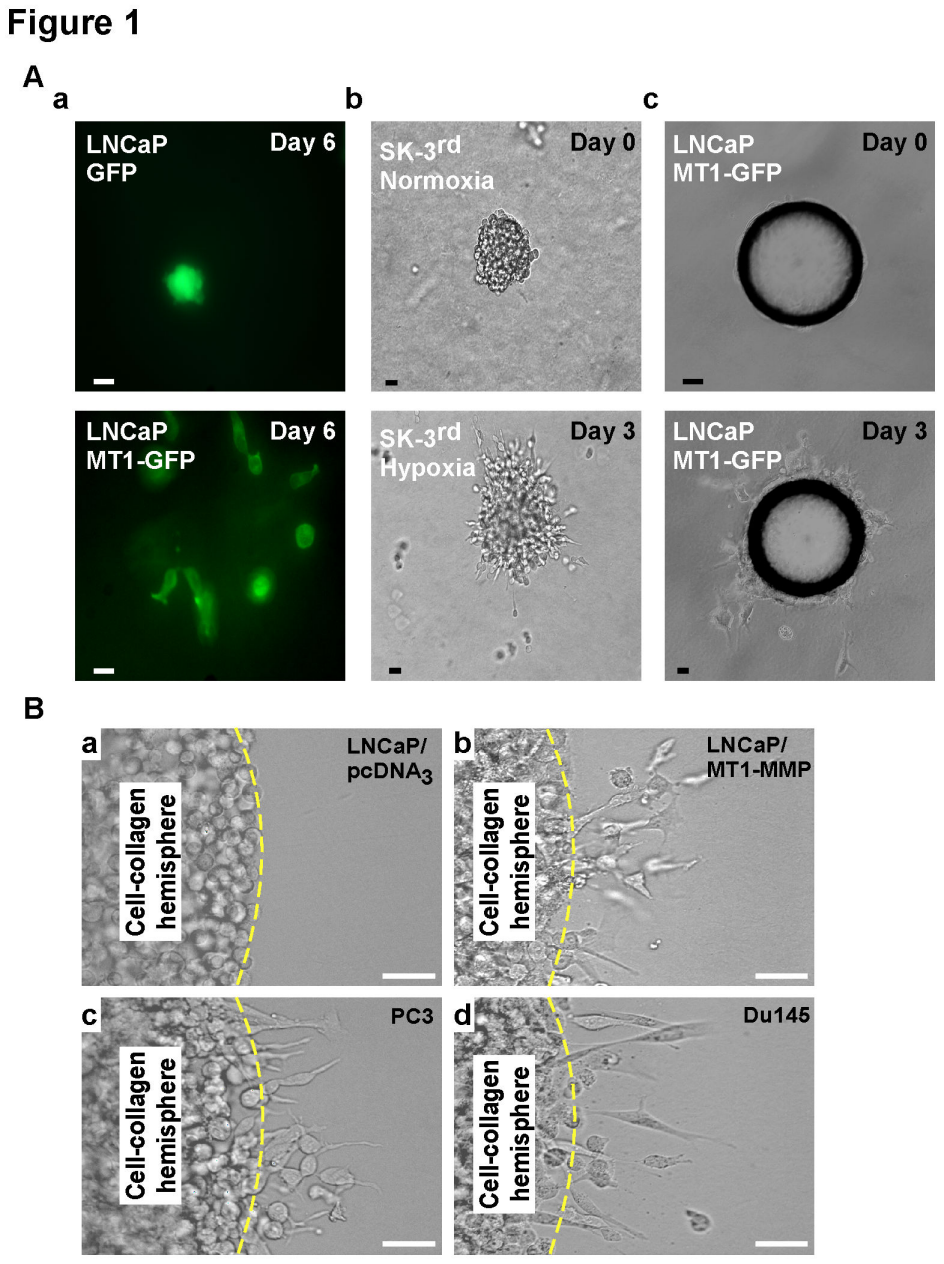
Cell scattering assays for monitoring cell invasive ability. A) a. Single-cell scattering assay: Single, isolated LNCaP cells were mixed with collagen and examined daily. MT1-GFP-expressing cells gradually displayed a scattering growth pattern after 6 days in culture as compared to the aggregated growth of GFP-expressing cells. b. Aggregate scattering assay: SK-3^rd^ cells were cultured for 12 days to form mammospheres, transferred and embedded in collagen, and then cultured under normoxia or hypoxia. A scattered pattern was seen after 3 days under hypoxia. c. Microcarrier bead scattering assay: MT1-GFP expressing LNCaP cells were cultured with beads for 24 hours, then transferred and embedded into collagen. Invaded cells were seen as a scattered pattern at day 3. Scale bars = 20 μm. B) Assessment of cancer cell invasiveness using novel 3-D invasion assay: LNCaP cells expressing vector control (a) or MT1-MMP (b), human prostate cancer PC3 cells (c), and Du145 cells (d) were suspended in collagen, dotted into the well of a 96-well plate, and covered with collagen followed by media. After 18-h incubation, PC3 and Du145, as well as MT1-MMP expressing LNCaP cells, showed increased numbers of invaded cells as compared to LNCaP vector control cells. Scale bar = 50 μm.

Currently used 3-D invasion assays are based on monitoring cell scattering ability and can be assembled in several ways. However, they all lack key attributes required for HT screening capability. For example, some assays cannot distinguish between inhibitors of cell proliferation and invasion. In these types of assays, single cells embedded in matrices, such as type I collagen, are examined for their invasive behavior based on their scattering growth pattern following proliferation. In Figure 1A-a, LNCaP cells, a less invasive human prostate cancer cell line, stably expressing membrane type 1 matrix metalloproteinase (MT1-MMP) fused with a green fluorescent protein (GFP) (MT1-GFP) displayed a scattering growth pattern, indicative of invasive behavior, as compared to GFP control cells that instead formed a cell aggregate within the gel. This is consistent with previous reports demonstrating that MT1-MMP can induce invasion of non-aggressive cell types[23]. However, both cell invasion and proliferation are required to form the scattered growth pattern and there is a lack of standardization hindering automated readout. Therefore, this single cell scattering assay is not suitable for HT anti-cancer drug discovery specifically aimed at targeting cancer invasion. 

In order to avoid these obstacles, the multi-cell spheroid method[20] was introduced, which has been recognized as having potential to be used as a HT invasion assay. However, as demonstrated through the use of SK-3^rd^ cells cultured under hypoxic conditions, which is known to increase the trafficking of MT1-MMP to the cell surface[22], spheroid formation to endpoint assessment of invasion requires long culture times, generally taking 6 to 12 days, and lacks size uniformity (Figure 1A-b). To avoid size variation, glass microcarrier beads with identical sizes can be utilized as scaffolds for spheroid formation[24], as shown in Figure 1A-c using LNCaP MT1-GFP expressing cells. However, the location of the beads containing cells after transferring to a matrix cannot be made uniform. Although these assays can be used for monitoring cancer cell invasion, long run times and a lack of standardization and automated readout capabilities hamper their utilization as HT screening tools.

### Establishment of a Quantitative 3-D invasion Assay for Evaluating Cancer Cell Invasion

Recognizing the need for a 3-D HT assay that tests the invasive ability of cells with a faster run time, we developed a quick collagen gel based 3-D invasion assay. To demonstrate the ability of our assay to detect differences in the invasive capacity of various cancer cell types, human prostate cancer cell lines with varying levels of aggressiveness, including androgen independent (Du145 and PC3) and dependent (LNCaP) cell lines as well as MT1-MMP expressing LNCaP cells, were examined. The indicated cancer cells were combined with neutralized native type I collagen, dotted into the center of each well of a 96-well plate, and allowed to solidify, ultimately forming a cell-collagen dot/hemisphere with a distinct boundary and shape. Following solidification, the cell-collagen hemispheres were embedded within a cover-layer of neutralized type I collagen and allowed to solidify. Media were then added to each well and the cells were allowed to invade into the surrounding matrix for 18 hours. As shown in Figure 1B, the metastatic Du145 and PC3 cells showed increased numbers of invasive cells protruding beyond the original cell-collagen boundary, whereas LNCaP cells expressing vector control cDNA failed to display cell invasion. Furthermore, the effect of MT1-MMP expression on cell invasion was easily determined by an increase in the number of invaded cells in MT1-MMP expressing LNCaP cells as compared to vector control cells (Fig. 1Ba & b). 

**Figure 2:**
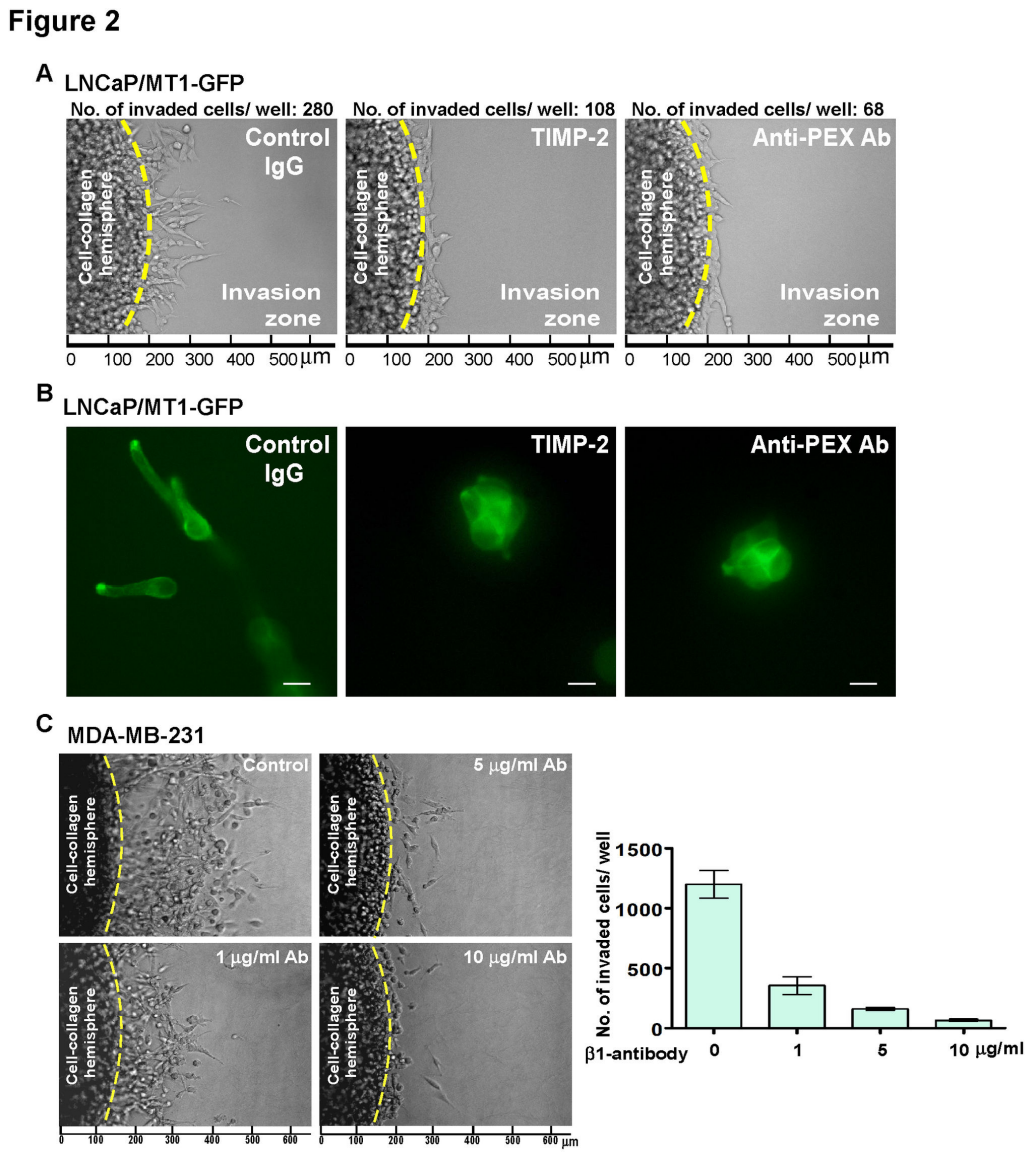
Evaluation of novel 3-D invasion assay. A & B) Comparison of the novel 3-D invasion assay and 3-D cell scattering assay: The effect of control IgG (Rabbit, 10 μg/ml), tissue inhibitor of metalloproteinase-2 (TIMP-2) (10 nM), or anti-MT1-MMP-hemopexin domain antibody (Anti-PEX Ab) (10 μg/ml) on MT1-GFP-induced LNCaP cell invasion was evaluated via the 3-D invasion assay (A) and 3-D cell scattering assay (B). An invasive growth pattern was photographed on day 1 (A) and day 6 (B) respectively. TIMP-2 and the anti-PEX Ab decreased the cell invasive/scattering ability of MT1-GFP expressing cells. Scale bar = 20 μm. C) Dose-dependent inhibition of human breast cancer MDA-MB-231 cells by anti-β1 integrin antibody: MDA-MB-231 cell invasion was examined using the 3-D invasion assay in the presence of the anti-β1 integrin antibody at different concentrations vs. control IgG. Invaded cells were microscopically examined (left panel) and counted (right panel). Bars represent the mean + SD.

In order to directly compare our novel 3-D invasion assay to the currently used 3-D cell scattering assay, the inhibitory effect of tissue inhibitor of metalloproteinase-2 (TIMP-2, an endogenous inhibitor of MT1-MMP)[25] and the anti-PEX antibody (directed against the MT1-MMP hemopexin domain)[26] on MT1-GFP induced LNCaP cell invasion were tested. For the novel 3-D invasion assay, the cells were set up as described for Figure 1B with the addition of either TIMP-2 or the anti-PEX antibody to the cell culture medium. Quantification of invaded cells revealed 61% and 76% reduction of MT1-GFP induced cell invasion by TIMP-2 and the anti-PEX antibody, respectively (Figure 2A). The 3-D cell scattering assay, shown in Figure 2B, qualitatively demonstrates inhibition of MT1-GFP-induced cell invasion via treatment with either TIMP-2 or the anti-PEX antibody. This comparison indicates that although these scattering assays provide clear, qualitative data demonstrating cancer cell invasion, they are not suitable for HT drug discovery programs that require quantitative results, which our assay can provide.

To determine the efficacy of our assay, a dose response test was performed using MDA-MB-231, an invasive breast cancer cell line, and an anti-β1 integrin antibody, which is known to inhibit cell migration, a key step for cancer cell invasion. A dose-dependent inhibition of MDA-MB-231 cell invasion upon treatment with the anti-β1 integrin antibody was easily quantified (Figure 2C). Collectively, these data demonstrate the ability of the novel 3-D invasion assay to easily, effectively, and quantitatively assess the invasive phenotype of various cancer cells as well as the effects of drugs on invasiveness.

### Standardization of the Developed Invasion Assay

**Figure 3:**
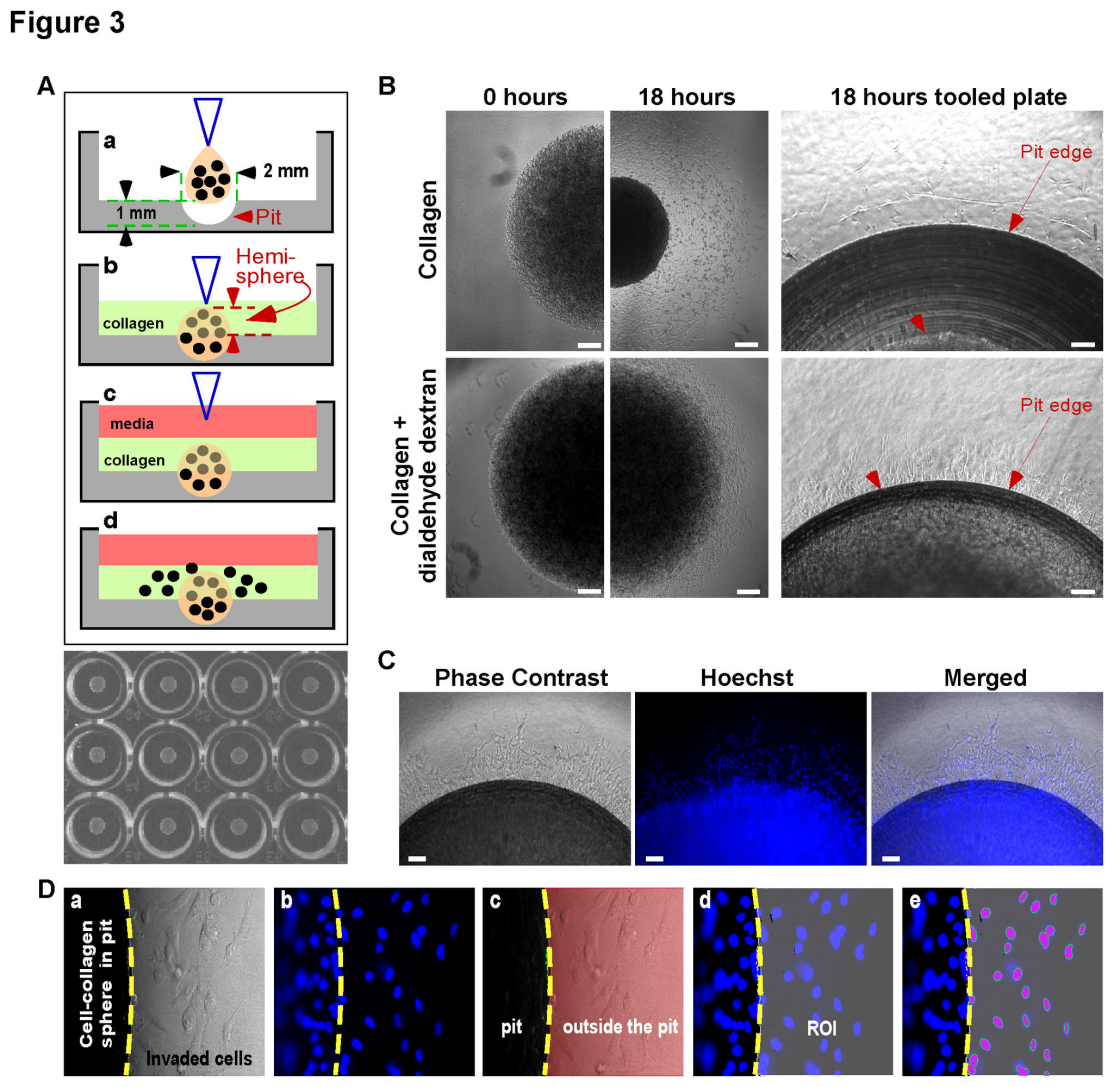
Standardization and automation of the 3-D invasion assay. A) Schematic diagram (top panel) of the 3-D invasion assay with tooled plate: (a) The cell-collagen mixture is dotted into the 2 mm in diameter pits in the center of each well of the tooled 96-well plate, creating spheres that occupy a volume of 4.18 mm^3^; (b) protruding cell-collagen hemispheres are covered with collagen; (c) medium containing compounds is added and plate is incubated for 18 hours; and (d) invaded cells beyond pit boundary are counted. Black dots represent cells. Diagram not to scale. An image of a section of a tooled plate with a pit in the center of each well is shown (bottom panel). B) Inhibition of collagen contraction via dialdehyde dextran: HT1080 cells were mixed with collagen with or without the addition of dialdehyde dextran before overlaying with collagen. Phage contrast images were taken at time 0 and 18 hours (left panel, Scale bar = 250 μm) and 18 hours in the tooled plate (right panel, Scale bar = 100 μm). Red arrows indicate the edge of the pits. Red arrowheads indicated the edge of the cell-collagen spheres. C) Phase contrast and fluorescent images of invaded HT1080 cells in the tooled plate stained with Hoechst dye. Scale bar = 100 μm. D) Automated quantification: Both phase contrast and fluorescent images (a & b) of HT1080 cells following invasion were acquired using a Nikon TE-2000s controlled by the NIS Elements imaging software. A threshold of the phase contrast image of the first well was adjusted based on the contrast to create two binary layers, one inside the pit (highlighted in black) and one outside of pit (highlighted in pink) (c). The binary layer outside the pit (pink area) was copied to form a region of interest (ROI), which was then applied to the Hoechst image (d). The invaded cells within the ROI were then automatically counted with the counted cells appearing purple in color (e).

As demonstrated, the most common obstacles in developing 3-D HT screens are standardization and automated readout. Although our assay is simple and fast, size homogeneity and placement of the cell-collagen hemisphere create a hurdle for these criteria. In order to standardize and allow for automation, a tooled 96-well plate with a 2 mm diameter pit (4.18 mm^3^) in the center of each well was designed (Figure 3A). The pit is of uniform size and location within each well, permitting standardization of the 3-D invasion assay and automated readout controlled by the NIS Elements (Nikon) imaging software along with a motorized stage.

A common limitation of the use of a collagen-based gel for 3-D cell culture is contraction due to remodeling of collagen fibrils by various cell types[27,28] that changes the overall size of the cell-collagen gel. To demonstrate this, HT1080 human fibrosarcoma cell-collagen hemispheres were imaged at time 0 and 18 hours. The cell-collagen hemispheres contracted, significantly decreasing the diameter from 0 to 18 hours and therefore preventing an accurate quantification of cell invasion beyond the parameter of the pit (Figure 3B, top panel). To overcome this, the addition of dialdehyde dextran, which has the ability to crosslink collagen[29], was assessed. Dialdehyde dextran was mixed with the HT1080 cell-collagen mixture before dotting into each well. As shown in Figure 3B (bottom panel), the addition of dialdehyde dextran significantly inhibited collagen contraction and maintained the original size and boundary of the cell-collagen hemispheres. No inhibitory effect on cancer cell invasion was observed. 

To determine if this novel design is capable of automated readout, a combination of the tooled invasion plate, an automated microscopic stage (Prior Scientific, Inc.), and imaging software (Nikon, NIS-Elements) was utilized. After 18 hours incubation, HT1080 cells were stained with the nuclear staining Hoechst dye followed by image acquisition. Both phase contrast and nuclear-stained images of HT1080 cells were taken for each well; a representative image is shown in Figure 3C. A threshold from the phase contrast image was adjusted based on the difference in contrast between inside the pit and outside the pit (the invasion area) to create binary layers. The defined binary layer outside the pit area was then copied to form a region of interest (ROI), which was then applied to the Hoechst image. The number of cells within the ROI, or invasion area, was then automatically counted (steps outlined in Fig. 3Da-e). Using the Nikon 10x (0.25 NA) objective lens and a motorized stage installed on a Nikon TE2000s inverted microscope, the scanning of an entire 96-well plate with 4 images per well could be completed within 6 minutes (data not shown). The ability to rapidly acquire and analyze data allows for the screening of many compounds in a short time frame, which is necessary for HT screenings. 

To determine the robustness, or Z factor[30], of our 3-D invasion assay, the effects of DMSO and the anti-β1 integrin antibody treatment on invasion of Du145 cells or MT1-GFP expressing LNCaP cells were tested. The Z factor was described by Zhang as Z factor=1-3xSSD/R (SSD: Sum of Standard Deviations; R: absolute value of the difference between positive and negative controls), with a Z factor value between 0.5 and 1.0 indicating that the assay is reliable[30]. Data collected on 5 different days were analyzed using the Z factor equation (data not shown). A Z-factor of 0.65 and 0.72 from Du145 cells and MT1-GFP expressing LNCaP cells, respectively, was calculated indicating that our 3-D invasion assay meets the criteria of an excellent assay for HT screening. Together, these data demonstrate the capability of our 3-D invasion assay to be used as a HT anti-invasive drug-screening tool.

### Simultaneous Determination of Compounds that Inhibit Cancer Cell Invasion from those that are Cytotoxic

**Figure 4:**
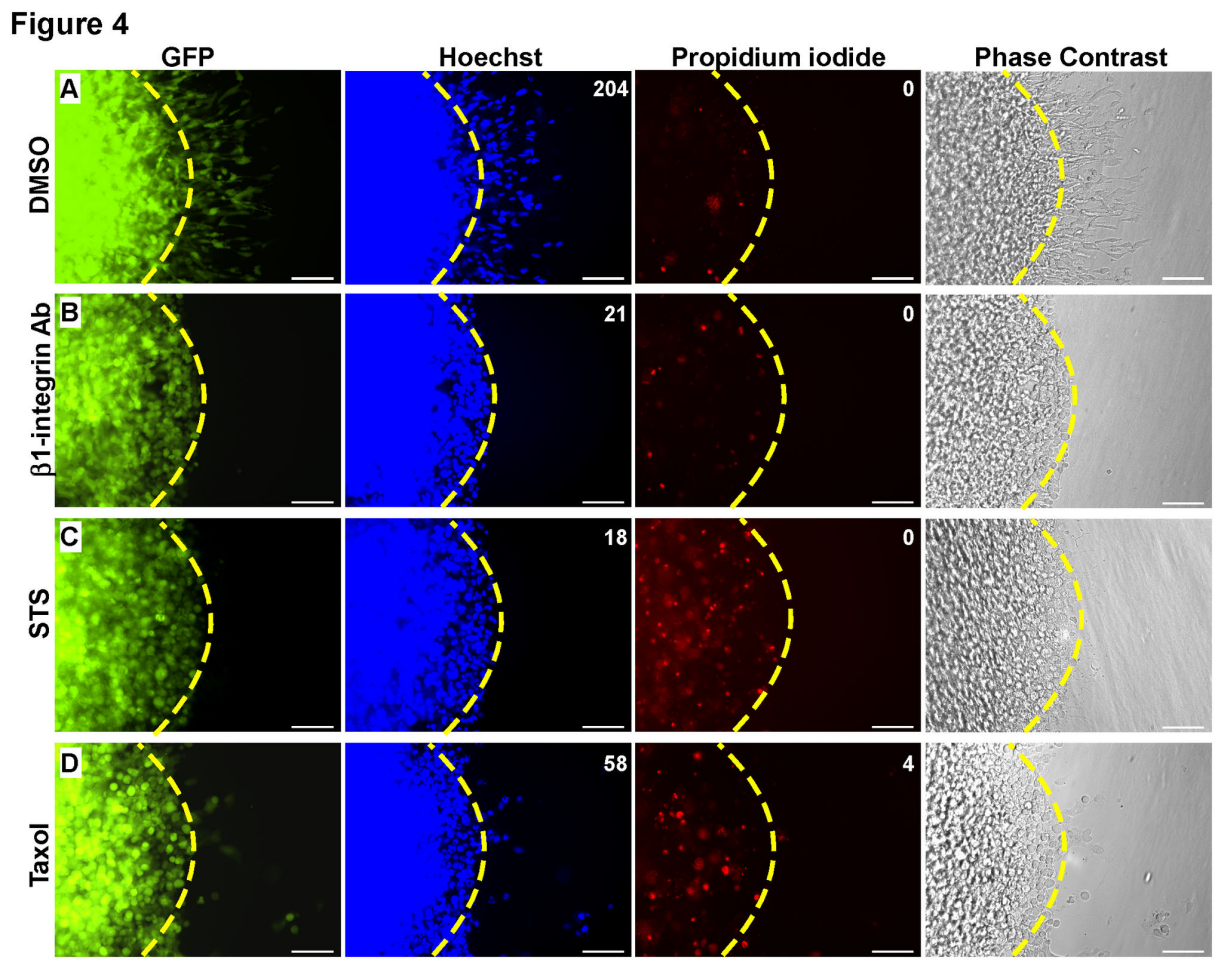
Simultaneous determination of invasion and cytotoxicity. HT1080 cells expressing GFP were assembled in the 3-D invasion assay in the presence of (A) DMSO, (B) anti-β1 integrin antibody (5 μg/ml), (C) staurosporine (STS) (500 nM), or (D) paclitaxel (Taxol, 1 μM) for 18 hours. The cells were stained with Hoechst and propidium iodide (PI) for 30 minutes followed by microscopic examination. Decreased cell invasion is observed upon treatment with anti-β1 integrin antibody, STS, and taxol. However, STS and taxol show higher levels of PI staining, indicative of increased cell death. Numbers in the top right corners represent the total number of invasive cells (Hoechst images) or cells that died after invading (PI images) within the field. Scale bar = 100 μm.

The ability to segregate between compounds that inhibit cancer cell invasion and those that induce cell death with the initial screen would circumvent the need for a secondary cytotoxicity screen, reducing overall cost and increasing efficiency. By co-staining the cell-collagen hemispheres with Hoechst and propidium iodide (PI), the total number of invaded cells and the cytotoxic effect of the drug can be simultaneously determined. To assess this approach, HT1080 cells expressing GFP cDNA were treated with DMSO, anti-β1 integrin antibody, staurosporine (STS), a kinase inhibitor known to induce apoptosis[31,32], or paclitaxel (taxol), a chemotherapeutic drug that stabilizes microtubules and inhibits cell division leading to cell death[33,34]. After 18 hours, the DMSO treated HT1080 GFP cells showed invasion into the surrounding collagen matrix with minimal PI staining, indicative of low cytotoxicity (Figure 4A). In contrast, cells treated with anti-β1 integrin antibody had decreased invasive ability but a similar level of PI staining (Figure 4B). Treatment with STS caused a significant increase in cell death, as evidenced by intense PI staining, and the cells displayed abrogated cell invasion (Figure 4C). Taxol, a slower acting drug, caused a significant reduction in cell invasion with a modest increase in cell death (Figure 4D). These data demonstrate the ability of our assay to segregate drugs that only inhibit cell invasion from drugs that block cancer cell invasion due to a cytotoxic effect. 

### Validation of the HT Capacity of the 3-D Invasion Assay

**Figure 5:**
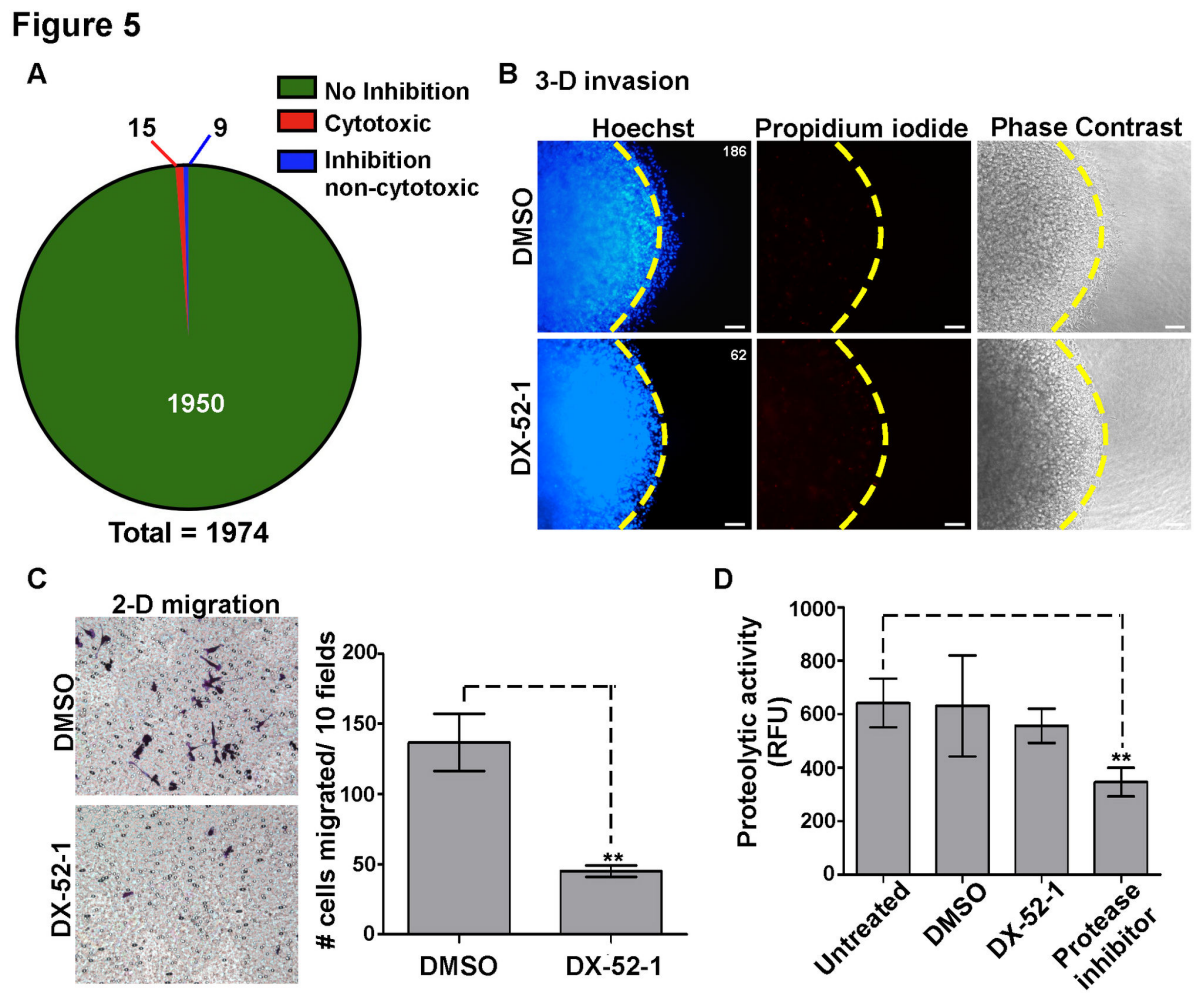
Primary screening using the developed 3-D invasion assay. A) The NCI Diversity Set II Compound Library was screened using the 3-D invasion assay and MDA-MB-231 cells, resulting in 24 positive hits, 9 of which were inhibitory but not cytotoxic. B) A quinocarmycin analog, DX-52-1 was positively identified as an anti-invasive compound. Representative images are shown for DMSO control and DX-52-1 (10 μM) after 18 hours incubation. Abrogated cell invasion and low cytotoxicity were observed in cells treated with DX-52-1. Numbers in the top right corners represent the total number of invasive cells within the field. Scale bar = 100 μm. C) Transwell chamber migration was performed with MDA-MB-231 cells treated with DMSO or DX-52-1 for 18 hours. Representative images of membranes (8 μm pore size) are shown in left panel. Quantification of cell migration is shown in right panel. DX-52-1 treated cells had decreased migratory ability. Bars represent the mean + SD. D) Fluorescent Protease Detection assay (Sigma) performed with cell lysates from MDA-MB-231 cells treated with DMSO or DX-52-1 for 18 hours. No effect on proteolytic activity was observed upon treatment with DX-52-1. Bars represent the mean + SD. ** refers to *p*<.01.

As a proof of principle, the National Cancer Institute Diversity Set II Compound Library, containing 1974 structurally diverse compounds, was screened using MDA-MB-231 breast cancer cell line. The compounds were added to the wells at a final concentration of 10 μM. DMSO, which is the vehicle, and anti-β1 integrin antibody were used as the negative and positive controls, respectively. Positive hits were determined based on a threshold of >50% inhibition of invasion. Based on this, 24 positive hits were found, 9 of which were confirmed to be <25% cytotoxic via a MTT assay (Figure 5A and data not shown). One of these hits was a known anti-migratory compound, the quinocarmycin analog DX-52-1[35], providing further proof that our assay can successfully identify compounds capable of inhibiting cancer cell invasion (Figure 5B). 

Due to the fact that target identification is helpful for predicting side effects and optimizing lead candidates, it is important to have downstream assays that can help elucidate the possible mechanism(s) of action of the positive hits. Since invasion requires both migratory and proteolytic activities, a vital next step is to distinguish between these two cellular properties. Utilizing DX-52-1 as an example, the conventional Transwell chamber migration assay and a non-specific protease assay (Sigma) were employed. As shown in Figure 5C & D, DX-52-1 significantly abrogated MDA-MB-231 cell migration but had no significant effect on protease activities. These data indicate that inhibition of the invasive behavior of the MDA-MB-231 cells by DX-52-1 is due to a reduction of migratory ability, which is supported by previously published reports[35]. Narrowing down the potential cellular processes affected by the compounds will focus future studies aimed at ascertaining the molecular target(s) of the drug.

## Discussion

Currently, two very different anti-cancer drug discovery approaches are being utilized in the pharmaceutical industry: target-specific and phenotypic-based[36]. Despite remarkable progress in target-based drug design and genomic, proteomic, and HT screening methods, the number of novel, single-target, FDA-approved cancer therapeutic drugs has fallen short of expectations [37]. The reason behind this unmet demand is in part due to a lack of biological efficacy and unfavorable pharmacokinetic properties, and more importantly, toxicity that is not addressed in primary target-based screens. Phenotypic screening, including the targeting of invasive behavior, can circumvent such problems. An obvious caveat is the lack of information on the drug target and/or mechanism of action, which limits the ability to predict off-target effects. However, new technologies are being developed to alleviate this issue[38] and the FDA is approving an increasing number of drugs prior to target identification. Furthermore, phenotypic screens have the potential to identify drugs that disrupt cell behavior through uncharacterized targets. Since metastasis accounts for 90% of treatment failure in patients with solid cancer, there is a pressing need for novel strategies to inhibit metastasis, regardless of which molecule is targeted.

Although there are numerous assays available for studying cancer cell invasion, none of them are currently suitable for HT screening. Drug screening programs rely on the ability to test and analyze many compounds in a rapid and reproducible manner, which requires an assay that is standardized and generates quantitative data. As demonstrated in Figure 1, previously established cell scattering assays require an extended period of time and only generate qualitative data. Furthermore, the multi-cell spheroid based methods are also technically challenging due to the requirement for transferring aggregates and they do not allow for easy standardization of size and placement, which hinders automated readout. Additionally, collagen contraction is another limitation among assays utilizing a collagen-based gel to measure invasion due to the change in shape and size of the gel. Overall, these limitations lower the potential for HT screening capabilities of current 3-D invasion assays. 

In this study, we demonstrate that our method not only requires minimal time and labor, but that the use of our tooled invasion plate along with the combination of collagen and dialdehyde dextran provide key requirements for standardization and automated quantification regardless of the cell type used. Dialdehyde dextran contains free aldehyde groups that are available to react with lysine residues of collagen. This cross-linking has been shown to harden various forms of hydrogels[29,39]. Additionally, dialdehyde dextran has been tested for use with various drug delivery systems and has shown no negative effects on cells tested[40,41]. Table 1 outlines 5 of the most commonly used 3-D invasion assays and highlights the advantages of our novel assay with respect to 4 essential qualities of any method designed for HT screening: rapid completion time, quantification, standardization, and automated data acquisition.

**Table 1:** Comparison of current 3-D invasions assay to novel 3-D HT invasion assay.

3-D invasion assays	Brief description of method	Assembly time	Quantification	Standardization	Automated readout
Cell Scattering[21]	Mixed cells in collagen matrix & check for cell scattering	1 hours	No	No	No
Cell Aggregates[20]	Aggregates formed via several ways then embedded	4-6 days	Yes	No	No
Microfluidic Assay[17]	Microchannels filled w/ matrices & allowed to gel; cells added	6 hours	Yes	No	No
Nested Collagen Matrices[19]	Contracted polymerized cell-matrix mixture placed on top of collagen, then covered w/ collagen	24 hours	Yes	No	No
Microcarrier Beads[24]	Cells coated onto beads; the coated beads mixed w/ matrices	1 day	Yes	No	No
3D HT Invasion	Cell-collagen mixture placed into pitted plate & covered w/ collagen	30 minutes	Yes	Yes	Yes	

This 3-D invasion assay also enables one-step analysis by simultaneously segregating truly inhibitory compounds from cytotoxic compounds, making this approach more economical and less complicated. Additionally, based on the results shown in Figure 1B, our assay has the potential to be utilized as a combined phenotypic- and target-based approach due to the ability to detect changes in invasive ability by a specific gene product. Therefore, it is applicable to investigating genes implicated in the process of metastasis and to identify compounds that target specific pro-metastatic genes; hence, this HT invasion assay has broad potential to bridge molecular biology and translational studies. Since cell invasive behavior also occurs in other pathological conditions[42,43], the current assay can be directly used for drug discovery in other diseases as well, e.g., atherosclerosis and various inflammatory diseases including rheumatoid arthritis. The implementation of our novel 3-D invasion assay into mainstream anti-cancer drug discovery programs will ultimately facilitate advancement in not only our general understanding of cancer cell invasion but also in our ability to better treat cancer dissemination, ultimately transforming cancer into a chronic disease. 
